# Oral Cancer Disparities in Low- and Middle-Income Countries: A Global Health Equity Perspective on Prevention, Early Detection, and Treatment Access

**DOI:** 10.5334/aogh.5003

**Published:** 2025-11-28

**Authors:** Delfin Lovelina Francis, Saravanan Sampoornam Pape Reddy

**Affiliations:** 1 Saveetha Dental College and Hospital, SIMATS, Saveetha University, Chennai, India; 2 Research and Referral Army Dental Corps, New Delhi, India

**Keywords:** cancer disparities, global health, india, oral cancer

## Abstract

*Background:* Oral cancer represents a critical global health equity challenge, with over 80% of cases occurring in low- and middle-income countries (LMICs) and markedly lower survival rates in these regions compared to high-income countries (HICs).

*Objective:* To examine oral cancer disparities in LMICs through a global health equity lens by analyzing prevention strategies, early detection programs, and treatment access barriers, with the aim of identifying evidence-based interventions to reduce these inequities.

*Methods:* This comprehensive narrative review synthesized evidence from peer-reviewed literature (2020–2025), including systematic reviews and reports from international health organizations. We searched PubMed, Web of Science, and Scopus using terms related to oral cancer, global health disparities, LMICs, prevention, screening, and treatment access.

*Results:* Oral cancer demonstrates profound global disparities. LMICs bear ~82% of the global disease burden yet achieve five-year survival rates of only 25–45%, compared to 65–85% in HICs. Key contributing disparities include the following: tobacco use remains high (LMICs account for 1.3 billion tobacco users) due to weak control programs. Limited human papillomavirus vaccination coverage is under 50% in most LMICs (vs. ~70–85% in HICs), and 70% of LMICs have no systematic oral cancer screening. In addition, 60–80% of oral cancer cases in LMICs present at advanced stages (vs. ~40–60% in HICs). They have severely limited access to surgery, radiotherapy, and chemotherapy (roughly 1 available service for every 5–10 needed).

*Conclusions:* Addressing oral cancer disparities in LMICs requires comprehensive strategies, including strengthened tobacco control, cost-effective screening programs using innovative technologies, task shifting to expand the health-care workforce, and international partnerships to improve treatment infrastructure in resource-poor settings. These combined efforts are essential to close the outcome gap and achieve global health equity in oral cancer care.

## Introduction

Oral cancer including malignancies of the lips, tongue, floor of the mouth, alveo-gingival complex, and adjacent subsites remains one of the most visible tests of global health equity. Although some high-income countries (HICs) still report higher age-standardized incidence due to historical tobacco and alcohol exposure, the overwhelming share of cases, fatality, and disability now occurs in low- and middle-income countries (LMICs), where prevention, early detection, and treatment capacity are constrained [1–3]. The consequence is a persistent survival gap, with LMICs experiencing markedly poorer outcomes despite the largely preventable nature of many tumors and the availability of cost-effective interventions [4]. India illustrates the paradox: a large share of the global burden, rising incidence and mortality, and entrenched risk behaviors that disproportionately affect poorer and rural populations [5].

This review uniquely integrates global evidence through a health equity framework to bridge epidemiological data with feasible implementation strategies in LMICs. It applies a global health equity lens to synthesize where and why disparities arise and how they can be reduced. We reviewed global burden and distribution, interrogated social and environmental determinants, and examined the health-system barriers that delay diagnosis and limit access to effective care. We then appraised the evidence for prevention, screening, and treatment strategies that are feasible in resource-constrained settings, emphasizing task sharing, digital tools, and regional cooperation, and concluded with a practical road map and policy priorities to accelerate equitable gains. Accordingly, we prioritize evidence that is scalable in LMIC contexts, trackable through routine indicators, and adaptable to local realities, with attention to gender, income, and geography. We also emphasize affordability, implementation quality, and accountability to communities. This review aims to critically analyze inequities in oral cancer burden, detection, and treatment between HICs and LMICs. It seeks to identify evidence-based determinants of disparity and policy levers to advance global oral cancer equity.

## Methods

**Figure 1:**
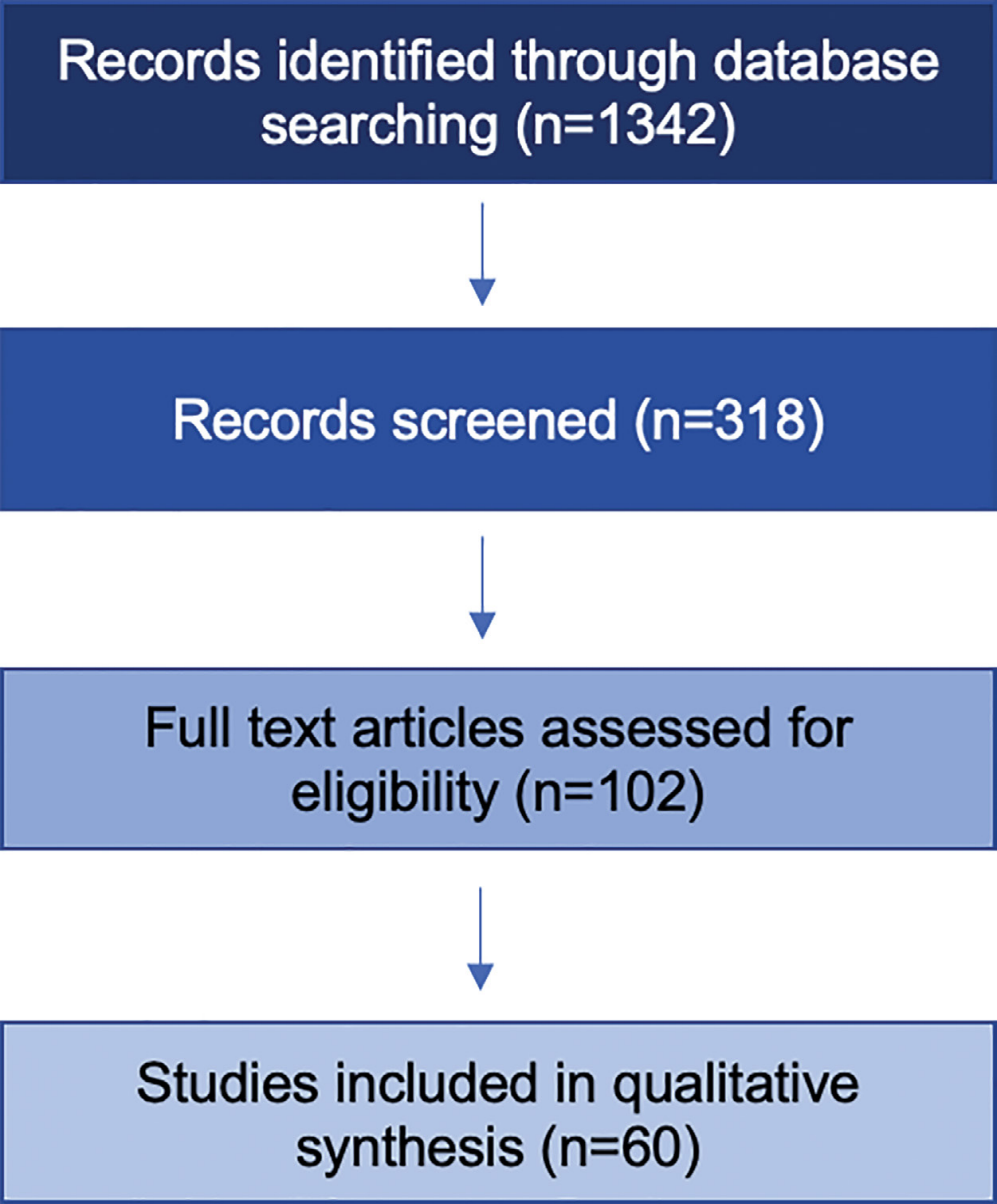
Methodology flowchart of the narrative review. The figure illustrates the structured approach used to identify, screen, and include studies in the qualitative synthesis (*n* = 60).

This narrative review followed the SANRA (Scale for the Assessment of Narrative Review Articles) quality framework to ensure methodological rigor. We conducted a structured search of PubMed, Scopus, and Web of Science for literature published between January 2020 and February 2025, using combinations of the following terms: “oral cancer,” “global health disparity,” “LMIC,” “prevention,” “screening,” and “treatment access.” English-language, peer-reviewed studies, systematic reviews, and global health reports addressing incidence, risk factors, screening, or treatment access in LMICs or comparative LMIC/HIC settings were included. Case reports, editorials, and studies unrelated to oral cancer or not containing country-level data were excluded (Figure 1). The extracted data were synthesized under four domains: (1) behavioral and environmental risk exposures, (2) screening and diagnostic readiness, (3) treatment access and health-system capacity, and (4) financial protection and policy implementation. All findings were analyzed comparatively between HIC and LMIC contexts based on World Bank income classification (2024).

## Global Burden and Determinants of Inequity

The distribution of oral cancer is geographically skewed. South and Southeast Asia bear a disproportionate burden; India alone contributes about one-third of global oral cancer cases despite accounting for only 18% of the world’s population, reflecting entrenched risk profiles and variable system readiness [6]. Incidence varies widely from ~1.9 per 100,000 in parts of sub-Saharan Africa to ~12.6 per 100,000 in South-Central Asia, but these figures likely underestimate true burden where diagnostic and registry capacity are also limited [7]. Mortality-to-incidence ratios (M:I) remain ~0.68 in LMICs versus ~0.43 in HICs, mirroring systemic constraints in access to diagnostics and care [8]. Registry coverage is especially sparse in Africa, with population-based registries capturing only a small fraction of cases, compounding uncertainty and hampering planning [9]. Within-country inequities add another layer, such as survival can differ five-fold between urban and rural areas in Brazil and indigenous communities experience mortality two to three times of national averages due to delayed diagnosis and treatment abandonment [10]. Social gradients are marked: poorer quintiles often face two- to four-fold higher incidence and three- to six-fold higher mortality than wealthier groups [14]. Demographic patterns also differ. Compared with HICs, LMICs diagnose oral cancer at younger ages often in the fifth rather than the seventh decade, consistent with early initiation and prolonged exposure to tobacco, areca, and alcohol [11]. Earlier onset amplifies socioeconomic impact since illness commonly affects primary earners during peak productive years and can precipitate long-term impoverishment [12]. Gender gaps, historically around 3–5:1 (male:female), are narrowing in some settings where women’s smokeless tobacco and betel quid use is increasing [13]. With the poorest and most remote communities facing the greatest exposure and the least access, inequity is produced and reproduced across generations [14].

### Survival and functional outcomes

Five-year survival underscores the equity divide where HICs report ~68–85% overall survival (with >90% for stage I–II), whereas LMICs often achieve only ~25–45% and some subregions fall below 20% [15]. Stage at presentation explains that much of the gap such as 55–70% is in early-stage detection in HICs versus 15–35% in LMICs, but differences persist even after adjusting for stage, reflecting systemic deficits in surgical, radiation, pathology, and supportive care [16, 17]. Post-treatment function and quality of life (speech, swallowing, and nutrition) are poorer in LMICs because the reconstructive options such as speech therapy and multidisciplinary rehabilitation are less available [18].

## Risk Factors and Social Determinants

### Tobacco (combusted and smokeless), areca, and alcohol

Tobacco remains the dominant risk factor. Over 80% of the world’s 1.3 billion tobacco users live in LMICs, where product diversity complicates regulation and cessation [19]. In addition to manufactured cigarettes, LMIC markets feature bidis and kreteks, water pipe products, and an array of smokeless forms, such as gutka, khaini, and zarda often mixed with areca nut and slaked lime, creating an alkaline milieu that augments mucosal carcinogen absorption [20, 21]. Toxicant profiles can exceed those of cigarettes; tobacco-specific nitrosamines in smokeless mixtures are sometimes several-fold higher; and sustained mucosal contact magnifies the exposure [22]. The summary of representative studies is presented in Table 1.

**Table 1:** Summary of representative publications.

AUTHOR (YEAR)	STUDY TYPE	JOURNAL	REGION/COUNTRY	KEY FINDINGS RELEVANT TO REVIEW
Sankaranarayanan et al. (2015)	Cluster RCT	*World Bank Data*	Global and LMICs	Community-based oral cancer screening reduced mortality by 34% among high-risk users.
Atun et al. (2015)	Policy analysis	*Lancet Oncol*	Global	Identified radiotherapy access gap of 65% in LMICs.
Mehrtash et al. (2017)	Consensus policy paper	*Lancet Oncol*	South Asia	Highlighted areca nut as emerging carcinogenic driver.
Gupta et al. (2016)	Epidemiological study	*Nepal J Epidemiol*	LMICs	Documented 1.3 billion tobacco users concentrated in LMICs.
Bray et al. (2024)	Epidemiologic report	*CA Cancer J Clin*	Global	Updated GLOBOCAN estimates showing >80% of oral cancer burden in LMICs.

Alcohol is a potent cofactor. Many LMICs have parallel markets for home-distilled or informally sold beverages with variable ethanol content and contaminants (e.g., methanol and acetaldehyde) that raise carcinogenic potential beyond commercial products [23]. Tobacco–alcohol synergy is multiplicative; cousers have ~10–20× higher oral cancer risk than abstainers [24]. Cultural norms varied from ceremonial use to perceived medicinal value which shape acceptability and complicate policy management, requiring locally tailored strategies [25].

### Environmental, occupational, and waterborne exposures

Structural exposures elevate baseline risk. Ambient air pollution exceeds WHO guidelines for ~92% of LMIC populations, while indoor biomass combustion exposes ~2.6 billion people to carcinogens such as benzopyrene and formaldehyde at concentrations far above outdoor urban levels in HICs [26, 27]. Occupational exposures such as pesticides in agriculture and solvents and dusts in industry are common under weak regulatory enforcement and limited personal protective equipment [28]. Chronic ingestion of toxic metals, notably arsenic in groundwater, affects over 140 million people globally, with concentrations in some regions far surpassing safety thresholds [29].

### Nutrition, micronutrients, and food safety

Dietary insufficiency and food insecurity shape susceptibility. Diets low in fruits and vegetables (and therefore antioxidants and fiber) but high in processed and preserved foods reduce mucosal resilience and immune surveillance [30]. Micronutrient deficiencies including folate, vitamin B₁₂, and iron are highly prevalent (20–70% in some LMIC cohorts) and can impair DNA repair, methylation balance, and host defense [31]. Traditional preservation methods (smoking, salting, and fermentation) can generate nitrosamines and polycyclic aromatic hydrocarbons; aflatoxin contamination in staple foods remains common in tropical climates without adequate storage [32, 33].

## Health-System Barriers Across the Care Continuum

### Primary-care readiness and access

Primary-care platforms are the linchpin for prevention and early detection but remain under-resourced. Health-worker density averages ~2.3 per 1,000 in LMICs versus ~12.8 in HICs, limiting capacity for community outreach, risk counseling, and routine oral examination [34]. Oral-health content in medical and nursing curricula is minimal (often ≤8 hours), leaving frontline clinicians unprepared for systematic oral screening or potential malignant disorder (PMD) management [35]. Facility assessments indicate that 60–80% of primary centers lack basic equipment for oral inspection; digital photography that would facilitate remote consultation is uncommon [36]. Distance and cost are major barriers, with rural populations traveling 25–50 km to reach facilities and transportation frequently costing a day’s wages [37].

### Specialist, diagnostic, and therapeutic capacity

Downstream services are thinly distributed. The density of oral and maxillofacial surgeons averages ~0.1 per 100,000 in LMICs (vs. ~2.5 in HICs), and entire countries may lack training programs [38]. Pathologist availability is ~10× lower than in HICs; biopsy turnaround frequently spans 4–12 weeks, delaying definitive management [39]. Radiotherapy capacity meets only ~35% of need overall; several regions lack radiotherapy unit within 1,000 km, producing wait lists that render some tumors inoperable by the time therapy begins [40]. Quality assurance systems are limited, with external quality control in fewer than one-third of laboratories and routine outcome auditing in fewer than one-fifth of cancer centers [41].

### Referral pathways, records, and patient support

Referral clarity and care coordination remain weak. An estimated 70–85% of primary providers lack explicit referral criteria for suspicious lesions; patients often receive incomplete instructions, and back-referrals are rare [42]. Paper records dominate in 80–90% of systems, and loss of information across levels of care is common; electronic records cover less than 10% in many LMICs versus ~85–95% in HICs [43]. Patient navigation is effective for adherence in HICs, which is available in less than 5% of LMIC cancer centers where treatment abandonment rates of 25–45% have been reported [44]. Collectively, these data underscore how structural inequities, not only behavioral risks, drive outcome disparities (Table 2).

**Table 2:** Comparative indicators: Oral cancer in HICs vs. LMICs.

INDICATOR	HICs	LMICs	PRIMARY DATA SOURCE
Five-year survival (%)	65–85%	25–45%	CONCORD-3, *Lancet*, 2018
Stage I–II detection rate (%)	55–70%	15–35%	Sankaranarayanan et al., 2015
Radiotherapy units per 1 million population	12–15%	1–2%	Atun et al., 2015
Tobacco use prevalence (%)	22%	45%	WHO GHO, 2024
National oral cancer screening coverage (%)	60–70%	<10%	WHO PEN, 2023
HPV vaccination coverage (%)	75–90%	<50%	Bruni et al., 2016
Health expenditure (% GDP)	8–10%	2–4%	World Bank, 2024

## Prevention and Early Detection: Evidence and Feasibility

### Comprehensive tobacco control and alcohol policy

Beyond summarizing known disparities, this review synthesizes actionable levers for governments, health systems, and international partners to accelerate equity in oral cancer outcomes. WHO framework convention on tobacco control aligned strategies, such as taxation, smoke-free laws, advertising bans, pictorial warnings, and cessation support, remain the most powerful levers for primary prevention [45]. Modeling and empirical evaluations suggest that robust implementation can reduce oral cancer incidence by 20–40% within 10–15 years. Excise taxes are especially potent; a 10% price increase typically reduces consumption by 4–8%, with the largest effects among youth and low-income groups; however, policies must anticipate substitution and illicit trade dynamics to sustain gains [46]. Smoke-free legislation reduces exposure and changes norms but depends on enforcement capacity; mass-media campaigns with culturally tailored messaging raise quit attempts and deter initiation at low cost per disability adjusted life year (DALY) averted [47, 48].

### Human papillomavirus (HPV) vaccination

HPV contribute substantially to oropharyngeal cancers in younger cohorts, and expanding vaccination offers a dual benefit alongside cervical cancer prevention. Coverage in most LMICs remains less than 50%, but school-based delivery can achieve 80–95% uptake when paired with community engagement and health-worker training. Rwanda’s program reached 93% coverage by integrating schools, primary care, and communication strategies [49, 50]. Economic analyses consistently show cost-effectiveness ($100–$500 per DALY averted), particularly with pooled procurement mechanisms [51].

### Nutrition and food safety

Population strategies to increase fruit and vegetable intake through subsidies, supply-chain improvements, and social marketing are associated with 40–60% risk reductions in observational syntheses [52]. In high-risk groups with documented deficiencies, targeted supplementation (e.g., folate and B-complex) has slowed PMD progression by ~30–50% in trials [53]. Food-safety interventions to reduce aflatoxin-improved drying/storage, biocontrol, and regulatory standards have reduced contamination by 50–80% in program settings and brought cross-cancer benefits [54].

### Screening and triage pathways

Visual inspection by trained oral health-care providers is a pragmatic foundation for early detection in high-prevalence settings. With standardized protocols and supportive supervision, programs achieve approximately 60–85% sensitivity and 85–95% specificity, with cost-effectiveness ratios around $200–$800 per DALY averted [55]. Complementary evidence shows that 20–40 hours of hands-on training with refreshers sustains skill retention (>80% at one year) and that external quality assurance improves sensitivity by 15–25% [56, 57]. Adjunctive technologies (e.g., vital staining and autofluorescence) may raise sensitivity in some contexts, but their incremental value depends on cost, maintenance, and operator skill [58]. Digital innovations can extend reach. Smartphone-enabled tele-mentoring and store-and-forward imaging have demonstrated 80–90% diagnostic concordance with in-person specialist assessment when images and metadata follow standardized protocols [59]. Early artificial intelligence algorithms approach specialist-level accuracy in controlled datasets; the next step is prospective validation, bias assessment and lesion types, and embedding within referral pathways with human oversight [60].

## Treatment Access and Service-Delivery Models

### Surgical services and task sharing

Even basic surgical capacity is insufficient in many LMICs. Workforce analyses suggest that LMICs collectively possess only ~20–40% of the specialized personnel needed for current demand, with training pipelines graduating fewer than 50 specialists per year across many countries [45]. Infrastructure deficits such as operating rooms, anesthesia, and ICU beds limit case complexity, and equipment shortages affect 70–90% of facilities attempting cancer surgery [46]. Perioperative mortality for major oral cancer resections is approximately 8–15% in LMIC settings versus 1–3% in high-resource centers, where international-standard resources and training exist and outcomes converge, demonstrating feasibility [47]. Pragmatic task sharing, including training general surgeons for early-stage resections under specialist oversight with clear referral thresholds, has shown acceptable results and can be scaled with tele-mentoring and standardized protocols [48].

### Radiotherapy access and regional cooperation

Radiotherapy is a critical bottleneck. Meeting current needs would require 5,000–8,000 additional machines (approximately $15–$25 billion capital investment), as existing capacity serves only 35–45% of indicated patients [49]. Regional centers of excellence and cross-border referral networks can partially close gaps, and pooled procurement and shared maintenance have reduced per-patient costs by 40–60% and improved uptime in West African pilots [50]. Tele-radiotherapy with remote planning and quality assurance under standardized protocols has achieved acceptable plan quality (85–95% meeting benchmarks) when linked with on-site technical support [51].

### Financial protection

Cancer care imposes severe financial strain. In many LMICs, direct medical costs for oral cancer equal 2–5 times of annual household income; 80–95% of affected families experience catastrophic health expenditure [52]. Indirect costs such as transport, lodging, and wage loss often match or exceed medical bills, especially in rural households where travel to tertiary centers is prolonged [53, 54]. Insurance coverage is sparse (15–35%) and frequently limited by co-pays and benefit caps [55]. Countries that expanded social health insurance (e.g., Thailand and Rwanda) cut out-of-pocket spending by 60–80% and increased treatment completion [56]. Targeted budget lines for cancer equivalent to 0.1–0.3% of national health expenditure have produced measurable improvements in access where sustained [57].

## Global Cooperation, Policy Alignment, and Research

### WHO frameworks and “best buys”

The WHO Global Action Plan for noncommunicable diseases (NCDs) embeds oral cancer control within broader prevention, and the WHO “Best Buy” package highlights tobacco and alcohol policies as highly cost-effective levers [58, 59]. Modeling suggests that comprehensive implementation could avert 25–40% of oral cancer cases at less than $150 per DALY averted, an unusually favorable ratio for cancer control. The WHO Package of Essential NCD Interventions (PEN) provides a standardized, primary-care adaptable oral screening protocol that has proven feasible when combined with training and quality assurance [60].

### Implementation research and capacity building

Persistent gaps concern delivery science rather than discovery. Priorities include (i) prospective evaluation of scaled visual screening with digital decision support and tele-consultation, (ii) hybrid effectiveness–implementation trials of culturally tailored cessation and alcohol-reduction packages, (iii) pragmatic trials of task sharing for early oral cancer surgery with tele-mentoring, and (iv) cost and equity evaluations of regional radiotherapy networks. North–South and South–South partnerships can accelerate technology transfer with open-source digital tools, simplified treatment guidelines, and shared training curricula, while building local bioengineering and biomedical technician capacity.

This review is limited by variability in regional data completeness and heterogeneity in oral cancer definitions across registries. The narrative nature of the synthesis carries a risk of selection and publication bias. Despite these limitations, triangulation of multiple international data sources provides a robust approximation of global oral cancer disparities.

## Conclusions

Oral cancer inequities reflect the cumulative effects of exposure, poverty, and system capacity, and they are solvable with sustained, coordinated action. By focusing on four evidence-based determinants, such as tobacco exposure, screening access, treatment capacity, and financial protection, this review clarifies where inequities between HICs and LMICs are most actionable. Strengthening these domains offers a realistic path to measurable global reduction in oral cancer mortality. Success also requires research that answers implementation questions, routine equity-disaggregated monitoring, and accountability for results across sectors. When these elements move together, LMICs can close the outcome gap and transform oral cancer from a marker of inequity into a measure of progress toward health justice and universal cancer care. Regular public reporting of equity-disaggregated progress metrics builds trust, enables course correction, and motivates sustained cross-sector investment from governments, donors, and civil society at national and subnational levels.

## References

[r1] Sung H, Ferlay J, Siegel RL, et al. Global cancer statistics 2020: GLOBOCAN estimates of incidence and mortality worldwide for 36 cancers in 185 Countries. CA Cancer J Clin. 2021;71(3):209–249. doi:10.3322/caac.21660. 33538338

[r2] Bray F, Laversanne M, Sung H, et al. Global cancer statistics 2022: GLOBOCAN estimates of incidence and mortality worldwide for 36 cancers in 185 countries. CA Cancer J Clin. 2024;74(3):229–263. doi:10.3322/caac.21834. 38572751

[r3] Wu J, Chen H, Liu Y, Yang R, An N. The global, regional, and national burden of oral cancer, 1990–2021: A systematic analysis for the Global Burden of Disease Study 2021. J Cancer Res Clin Oncol. 2025;151(2):53. doi:10.1007/s00432-025-06098-w. PMC11775039 39875744

[r4] Warnakulasuriya S, Kerr AR. Oral cancer screening: Past, present, and future. J Dent Res. 2021;100(12):1313–1320. doi:10.1177/00220345211014795. 34036828 PMC8529297

[r5] Gupta N, Gupta R, Acharya AK, et al. Changing trends in oral cancer—a global scenario. Nepal J Epidemiol. 2016;6(4):613–619. doi:10.3126/nje.v6i4.17255. 28804673 PMC5506386

[r6] Shield KD, Ferlay J, Jemal A, et al. The global incidence of lip, oral cavity, and pharyngeal cancers by subsite in 2012. CA Cancer J Clin. 2017;67(1):51–64. doi:10.3322/caac.21384. 28076666

[r7] Ferlay J, Colombet M, Soerjomataram I, et al. Cancer statistics for the year 2020: An overview. Int J Cancer. 2021;149(4):778–789. doi:10.1002/ijc.33588. 33818764

[r8] Allemani C, Matsuda T, Di Carlo V, et al. Global surveillance of trends in cancer survival 2000-14 (CONCORD-3): Analysis of individual records for 37 513 025 patients diagnosed with one of 18 cancers from 322 population-based registries in 71 countries. Lancet. 2018;391(10125):1023–1075. doi:10.1016/S0140-6736(17)33326-3. 29395269 PMC5879496

[r9] Bray F, Parkin DM, African Cancer Registry Network. Cancer in sub-Saharan Africa in 2020: A review of current estimates of the national burden, data gaps, and future needs. Lancet Oncol. 2022;23(6):719–728. doi:10.1016/S1470-2045(22)00270-4. 35550275

[r10] Ramos LF, Sobrinho AR, Ribeiro LN, et al. Racial disparity and prognosis in patients with mouth and oropharynx cancer in Brazil. Med Oral Patol Oral Cir Bucal. 2022;27(4):e392–e396. doi:10.4317/medoral.25334. 35368007 PMC9271347

[r11] Johnson NW, Jayasekara P, Amarasinghe AA. Squamous cell carcinoma and precursor lesions of the oral cavity: Epidemiology and aetiology. Periodontol. 2000. 2011;57(1):19–37. doi:10.1111/j.1600-0757.2011.00401.x. 21781177

[r12] Knaul FM, Farmer PE, Krakauer EL, et al. Alleviating the access abyss in palliative care and pain relief—an imperative of universal health coverage: The Lancet Commission report. Lancet. 2018;391(10128):1391–1454. doi:10.1016/S0140-6736(17)32513-8. 29032993

[r13] Petersen PE. The World Oral Health Report 2003: Continuous improvement of oral health in the 21st century—the approach of the WHO Global Oral Health Programme. Community Dent Oral Epidemiol. 2003;31(Suppl 1):3–23. doi:10.1046/j..2003.com122.x. 15015736

[r14] Sankaranarayanan R, Ramadas K, Amarasinghe H, Subramanian S, Johnson N. Oral cancer: Prevention, early detection, and treatment. In: Gelband H, Jha P, Sankaranarayanan R, Horton S, eds. Cancer: Disease Control Priorities, Third Edition (Volume 3). The International Bank for Reconstruction and Development/The World Bank; 2015. 26913350

[r15] Atun R, Jaffray DA, Barton MB, et al. Expanding global access to radiotherapy. Lancet Oncol. 2015;16(10):1153–1186. doi:10.1016/S1470-2045(15)00222-3. 26419354

[r16] Farmer P, Frenk J, Knaul FM, et al. Expansion of cancer care and control in countries of low and middle income: A call to action. Lancet. 2010;376(9747):1186–1193. doi:10.1016/S0140-6736(10)61152-X. 20709386

[r17] Sullivan R, Alatise OI, Anderson BO, et al. Global cancer surgery: Delivering safe, affordable, and timely cancer surgery. Lancet Oncol. 2015;16(11):1193–1224. doi:10.1016/S1470-2045(15)00223-5. 26427363

[r18] Gupta B, Johnson NW. Emerging and established global life-style risk factors for cancer of the upper aero-digestive tract. Asian Pac J Cancer Prev. 2014;15(15):5983–5991. doi:10.7314/apjcp.2014.15.15.5983. 25124561

[r19] Shankar A, Parascandola M, Sakthivel P, Kaur J, Saini D, Jayaraj NP. Advancing tobacco cessation in LMICs. Curr Oncol. 2022;29(12):9117–9124. doi:10.3390/curroncol29120713. 36547127 PMC9777415

[r20] Mehrtash H, Duncan K, Parascandola M, et al. Defining a global research and policy agenda for betel quid and areca nut. Lancet Oncol. 2017;18(12):e767–e775. doi:10.1016/S1470-2045(17)30460-6. 29208442

[r21] Itumalla R, Khatib MN, Gaidhane S, et al. Smokeless tobacco consumption among women of reproductive age: A systematic review and meta-analysis. BMC Public Health. 2024;24(1):1361. doi:10.1186/s12889-024-18840-z. PMC11106917 38769491

[r22] Bushi G, Khatib MN, Balaraman AK, et al. Prevalence of dual use of combustible tobacco and E-cigarettes among pregnant smokers: A systematic review and meta-analysis. BMC Public Health. 2024;24:3200. doi:10.1186/s12889-024-20746-9. 39558300 PMC11572542

[r23] Bagnardi V, Rota M, Botteri E, et al. Alcohol consumption and site-specific cancer risk: A comprehensive dose-response meta-analysis. Br J Cancer. 2015;112(3):580–593. doi:10.1038/bjc.2014.579. 25422909 PMC4453639

[r24] Hashibe M, Brennan P, Chuang SC, et al. Interaction between tobacco and alcohol use and the risk of head and neck cancer: Pooled analysis in the International Head and Neck Cancer Epidemiology Consortium. Cancer Epidemiol Biomarkers Prev. 2009;18(2):541–550. doi:10.1158/1055-9965.EPI-08-0347. 19190158 PMC3051410

[r25] Room R, Babor T, Rehm J. Alcohol and public health. Lancet. 2005;365(9458):519–530. doi:10.1016/S0140-6736(05)17870-2. 15705462

[r26] Lim SS, Vos T, Flaxman AD, et al. A comparative risk assessment of burden of disease and injury attributable to 67 risk factors and risk factor clusters in 21 regions, 1990-2010: A systematic analysis for the Global Burden of Disease Study 2010. Lancet. 2012;380(9859):2224–2260. doi:10.1016/S0140-6736(12)61766-8. 23245609 PMC4156511

[r27] Gordon SB, Bruce NG, Grigg J, et al. Respiratory risks from household air pollution in low and middle income countries. Lancet Respir Med. 2014;2(10):823–860. doi:10.1016/S2213-2600(14)70168-7. 25193349 PMC5068561

[r28] Global Burden of Disease 2019 Cancer Collaboration, Kocarnik JM, Compton K, et al. Cancer incidence, mortality, years of life lost, years lived with disability, and disability-adjusted life years for 29 cancer groups from 2010 to 2019: A systematic analysis for the global burden of disease study 2019. JAMA Oncol. 2022;8(3):420–444. doi:10.1001/jamaoncol.2021.6987. 34967848 PMC8719276

[r29] Chowdhury R, Ramond A, O’Keeffe LM, et al. Environmental toxic metal contaminants and risk of cardiovascular disease: Systematic review and meta-analysis. BMJ. 2018;362:k3310. doi:10.1136/bmj.k3310. 30158148 PMC6113772

[r30] Pavia M, Pileggi C, Nobile CG, Angelillo IF. Association between fruit and vegetable consumption and oral cancer: A meta-analysis of observational studies. Am J Clin Nutr. 2006;83(5):1126–1134. doi:10.1093/ajcn/83.5.1126. 16685056

[r31] Black RE, Victora CG, Walker SP, et al. Maternal and child undernutrition and overweight in low-income and middle-income countries. Lancet. 2013;382(9890):427–451. doi:10.1016/S0140-6736(13)60937-X. 23746772

[r32] Santarelli RL, Pierre F, Corpet DE. Processed meat and colorectal cancer: A review of epidemiologic and experimental evidence. Nutr Cancer. 2008;60(2):131–144. doi:10.1080/01635580701684872. 18444144 PMC2661797

[r33] Liu Y, Wu F. Global burden of aflatoxin-induced hepatocellular carcinoma: A risk assessment. Environ Health Perspect. 2010;118(6):818–824. doi:10.1289/ehp.0901388. 20172840 PMC2898859

[r34] Crider K, Williams J, Qi YP, et al. Folic acid supplementation and malaria susceptibility and severity among people taking antifolate antimalarial drugs in endemic areas. Cochrane Database Syst Rev. 2022;2(2022):CD014217. doi:10.1002/14651858.CD014217. 36321557 PMC8805585

[r35] Petersen PE, Bourgeois D, Ogawa H, Estupinan-Day S, Ndiaye C. The global burden of oral diseases and risks to oral health. Bull World Health Organ. 2005;83(9):661–669. 16211157 PMC2626328

[r36] Watt RG, Daly B, Allison P, et al. Ending the neglect of global oral health: Time for radical action. Lancet. 2019;394(10194):261–272. doi:10.1016/S0140-6736(19)31133-X. 31327370

[r37] Penchansky R, Thomas JW. The concept of access: Definition and relationship to consumer satisfaction. Med Care. 1981;19(2):127–140. doi:10.1097/00005650-198102000-00001. 7206846

[r38] Yip W, Hsiao WC. Non-evidence-based policy: How effective is China’s new cooperative medical scheme in reducing medical impoverishment? Soc Sci Med. 2009;68(2):201–209. doi:10.1016/j.socscimed.2008.09.066. 19019519

[r39] Ruhangaza D, Kennedy LS, Tsongalis GJ. Providing diagnostic pathology services in low and middle-income countries. Hematol Oncol Clin North Am. 2024;38(1):209–216. doi:10.1016/j.hoc.2023.05.015. 37328312

[r40] Abdel-Wahab M, Bourque JM, Pynda Y, et al. Status of radiotherapy resources in Africa: An international atomic energy agency analysis. Lancet Oncol. 2013;14(4):e168–175. doi:10.1016/S1470-2045(12)70532-6. 23561748

[r41] Smith SM, Eadara A, Parkash V. Addressing quality and safety in anatomic pathology in low- and middle-income countries. Front Med (Lausanne). 2022;9:1060179. doi:10.3389/fmed.2022.1060179. 36619634 PMC9817141

[r42] Mars M, Scott RE. Global e-health policy: A work in progress. Health Aff (Millwood). 2010;29(2):237–243. doi:10.1377/hlthaff.2009.0945. 20348067

[r43] Kruse CS, Karem P, Shifflett K, Vegi L, Ravi K, Brooks M. Evaluating barriers to adopting telemedicine worldwide: A systematic review. J Telemed Telecare. 2018;24(1):4–12. doi:10.1177/1357633X16674087. 29320966 PMC5768250

[r44] Freeman HP, Muth BJ, Kerner JF. Expanding access to cancer screening and clinical follow-up among the medically underserved. Cancer Pract. 1995;3(1):19–30. 7704057

[r45] Tauras JA. Tobacco control in low-income and middle-income countries: Findings from WHO FCTC investment cases. Tob Control. 2024;33(Suppl 1):s1–s2. doi:10.1136/tc-2024-058717. 38697657 PMC11103319

[r46] Chaloupka FJ, Yurekli A, Fong GT. Tobacco taxes as a tobacco control strategy. Tob Control. 2012;21(2):172–180. doi:10.1136/tobaccocontrol-2011-050417. 22345242

[r47] Callinan JE, Clarke A, Doherty K, Kelleher C. Legislative smoking bans for reducing secondhand smoke exposure, smoking prevalence and tobacco consumption. Cochrane Database Syst Rev. 2010;(4):CD005992. doi:10.1002/14651858.CD005992.pub2. 20393945

[r48] Wakefield MA, Loken B, Hornik RC. Use of mass media campaigns to change health behaviour. Lancet. 2010;376(9748):1261–1271. doi:10.1016/S0140-6736(10)60809-4. 20933263 PMC4248563

[r49] Bruni L, Diaz M, Barrionuevo-Rosas L, et al. Global estimates of human papillomavirus vaccination coverage by region and income level: A pooled analysis. Lancet Glob Health. 2016;4(7):e453–e463. doi:10.1016/S2214-109X(16)30099-7. 27340003

[r50] LaMontagne DS, Bloem PJN, Brotherton JML, Gallagher KE, Badiane O, Ndiaye C. Progress in HPV vaccination in low- and lower-middle-income countries. Int J Gynaecol Obstet. 2017;138(Suppl 1):7–14. doi:10.1002/ijgo.12186. 28691329

[r51] Campos NG, Sharma M, Clark A, et al. The health and economic impact of scaling cervical cancer prevention in 50 low- and lower-middle-income countries. Int J Gynaecol Obstet. 2017;138(Suppl 1):47–56. doi:10.1002/ijgo.12184. 28691334

[r52] Boeing H, Bechthold A, Bub A, et al. Critical review: Vegetables and fruit in the prevention of chronic diseases. Eur J Nutr. 2012;51(6):637–663. doi:10.1007/s00394-012-0380-y. 22684631 PMC3419346

[r53] Garewal HS, Schantz S. Emerging role of beta-carotene and antioxidant nutrients in prevention of oral cancer. Arch Otolaryngol Head Neck Surg. 1995;121(2):141–144. doi:10.1001/archotol.1995.01890020005002. 7840919

[r54] Mitchell NJ, Bowers E, Hurburgh C, Wu F. Potential economic losses to the US corn industry from aflatoxin contamination. Food Addit Contam Part A Chem Anal Control Expo Risk Assess. 2016;33(3):540–550. doi:10.1080/19440049.2016.1138545. 26807606 PMC4815912

[r55] Sankaranarayanan R, Ramadas K, Thomas G, et al. Effect of screening on oral cancer mortality in Kerala, India: A cluster-randomised controlled trial. Lancet. 2005;365(9475):1927–1933. doi:10.1016/S0140-6736(05)66658-5. 15936419

[r56] Brocklehurst P, Kujan O, O’Malley LA, Ogden G, Shepherd S, Glenny AM. Screening programmes for the early detection and prevention of oral cancer. Cochrane Database Syst Rev. 2013;2013(11):CD004150. doi:10.1002/14651858.CD004150. 24254989 PMC8078625

[r57] Walsh T, Liu JL, Brocklehurst P, et al. Clinical assessment to screen for the detection of oral cavity cancer and potentially malignant disorders in apparently healthy adults. Cochrane Database Syst Rev. 2013;2013(11):CD010173. doi:10.1002/14651858.CD010173.pub2. 24258195 PMC7087434

[r58] Siddiqui AA, Abideen MZU, Abdullah M, et al. Investigating oral cancer awareness in outpatient settings: A hospital-based study. Bangladesh J Med Sci. 2025;24(2):450–456. doi:10.3329/bjms.v24i2.81712.

[r59] Elmore JG, Armstrong K, Lehman CD, Fletcher SW. Screening for breast cancer. JAMA. 2005;293(10):1245–256. doi:10.1001/jama.293.10.1245. 15755947 PMC3149836

[r60] Kujan O, Glenny AM, Duxbury J, Thakker N, Sloan P. Evaluation of screening strategies for improving oral cancer mortality: A Cochrane systematic review. J Dent Educ. 2005;69(2):255–265. 15689610

